# Flavonoid Constituents and Alpha-Glucosidase Inhibition of *Solanum stramonifolium* Jacq. Inflorescence with In Vitro and In Silico Studies

**DOI:** 10.3390/molecules27238189

**Published:** 2022-11-24

**Authors:** Sukanya Dej-adisai, Oraphan Sakulkeo, Chatchai Wattanapiromsakul, Thanet Pitakbut

**Affiliations:** 1 Department of Pharmacognosy and Pharmaceutical Botany, Faculty of Pharmaceutical Sciences, Prince of Songkla University, Hat Yai, Songkhla 90112, Thailand; 2 Technical Biochemistry, Department of Biochemical and Chemical Engineering, Technical University of Dortmund, 44227 Dortmund, Germany; 3 Pharmaceutical Biology, Department of Biology, Friedrich-Alexander-Universität Erlangen-Nürnberg (FAU), Staudtstr. 5, 91058 Erlangen, Germany

**Keywords:** *Solanum stramonifolium*, flavonoids, phytochemistry, alpha-glucosidase inhibition, molecular docking

## Abstract

*Solanum stramonifolium* Jacq. (Solanaceae) is widely found in South East Asia. In Thailand, it is used as vegetable and as a component in traditional recipes. The results of an alpha-glucosidase inhibitory screening test found that the crude extract of *S. stramonifolium* inflorescence exhibited the potential effect with IC_50_ 81.27 μg/mL. The separation was performed by the increasing solvent polarity method. The ethyl acetate, ethanol, and water extracts of *S. stramonifolium* inflorescence showed the synergistic effect together with acarbose standard. The phytochemical investigation of these extracts was conducted by chromatographic and spectroscopic techniques. Six flavonoid compounds, myricetin 3, 4′, 5′, 7-tetramethyl ether (**1**), combretol (**2**), kaempferol (**3**), kaempferol 7-*O*-glucopyranoside (**4**), 5-hydroxy 3-7-4′-5′-tetramethoxyflavone-3′-*O*-glucopyranoside (**5**), and a mixture (**6**) of isorhamnetin 3-*O*-glucopyranoside (**6a**) and astragalin (**6b**) were isolated. This discovery is the first report of flavonoid-glycoside **5**. Moreover, the selected flavonoids, kaempferol and astragalin, were representatives to explore the mechanism of action. Both of them performed mixed-type inhibition. The molecular docking gave a better understanding of flavonoid compounds’ ability to inhibit the alpha-glucosidase enzyme.

## 1. Introduction

Diabetes is a metabolic disease that is accompanied by hyperglycemia. In 2021, there were 537 million diabetic adults, and the number will increase to over 750 million by 2045 [1]. There are several classes of drugs used for controlling postprandial hyperglycemia. Alpha-glucosidase inhibitors are one of the most efficient therapeutic drug approaches to diabetes [2]. Therefore, this research focused on the anti-alpha-glucosidase model. Some researchers have suggested that using a combination drug may be beneficial over a single treatment due to the synergistic therapeutic effect, dose, and toxicity reduction [3]. A previous study showed an interesting result of the combination drug with a natural compound against alpha-glucosidase [4]. Moreover, the docking technique is a useful tool for the understanding of molecular interaction between the compound and the target protein, including the alpha-glucosidase enzyme [5].

The plants in the *Solanum* genus are wildly used in food and traditional medicines in various countries, including Thailand [6,7]. The *Solanum* plants, such as *S. nigrum*, *S. torvum*, *S trilobatum*, *S. sisymbrifolium*, *S. lycopersicum,* and *S. mammosum*, have been reported to have medicinal uses and phytochemicals [7]. From our preliminary screening of the alpha-glucosidase inhibition of *Solanum* species, the extract of *S. stramonifolium* inflorescence showed a strong inhibition, with 97.98 ± 0.64% at 2 mg/mL and half maximal inhibitory concentration (IC_50_) as 81.27 μg/mL. There is already a review of the anti-diabetic potential effects of the genus *Solanum* [8]. However, the study of *S. stramonifolium* has less information on the alpha-glucosidase enzyme; in particular, the inflorescence has not been reported. Thus, from the screening test, *S. stramonifolium* inflorescence was selected for the further investigation of chemical constituents, alpha-glucosidase inhibition, combination, mode of action, and molecular docking studies.

## 2. Results

### 2.1. Screening of Biological Activity and Chemical Constituents of S. stramonifolium Extracts

#### 2.1.1. Alpha-Glucosidase Inhibition

The screening of the alpha-glucosidase inhibitory activity was carried out, and the percentage inhibition values at 2 mg/mL and the IC_50_ values were used to classify the methanolic extracts from root, stem, leaves, and inflorescence of *S. stramonifolium*. Among these extracts, the inflorescence extract (SSIE) exhibited the highest percentage inhibition (97.98 ± 0.64%) and a good IC_50_ value (81.27 µg/mL), as shown in Table 1. Thus, the inflorescence of *S. stramonifolium* was selected for further investigation in this study.

The dried inflorescence of *S. stramonifolium* was extracted by increasing solvent polarity. Four extracts, including *n*-hexane extract (SSHX), EtOAc extract (SSEA), EtOH extract (SSEO), and boiled water extract (SSWT), were evaluated for their alpha-glucosidase inhibition, as shown in Table 1. The SSEA exhibited the highest potential alpha-glucosidase inhibition, with IC_50_ 215.92 µg/mL, while the acarbose standard showed an IC_50_ of 241.40 µg/mL.

#### 2.1.2. Total Phenolic Contents (TPC) and Total Flavonoid Contents (TFC)

In the previous review, flavonoid compounds were investigated in *Solanum* species and were reported as the bioactive compounds in this species [9]. Therefore, all extracts of *S. stramonifolium* inflorescence were evaluated to find the TPC and TFC. The results (as presented in Table 1) revealed that SSIE showed the highest amount of both TPC and TFC, followed by SSEO, SSWT, SSEA, and SSHX.

### 2.2. Combination Inhibitory Effect of S. stramonifolium Extracts and Acarbose on Alpha-Glucosidase Activity

**Figure 1:**
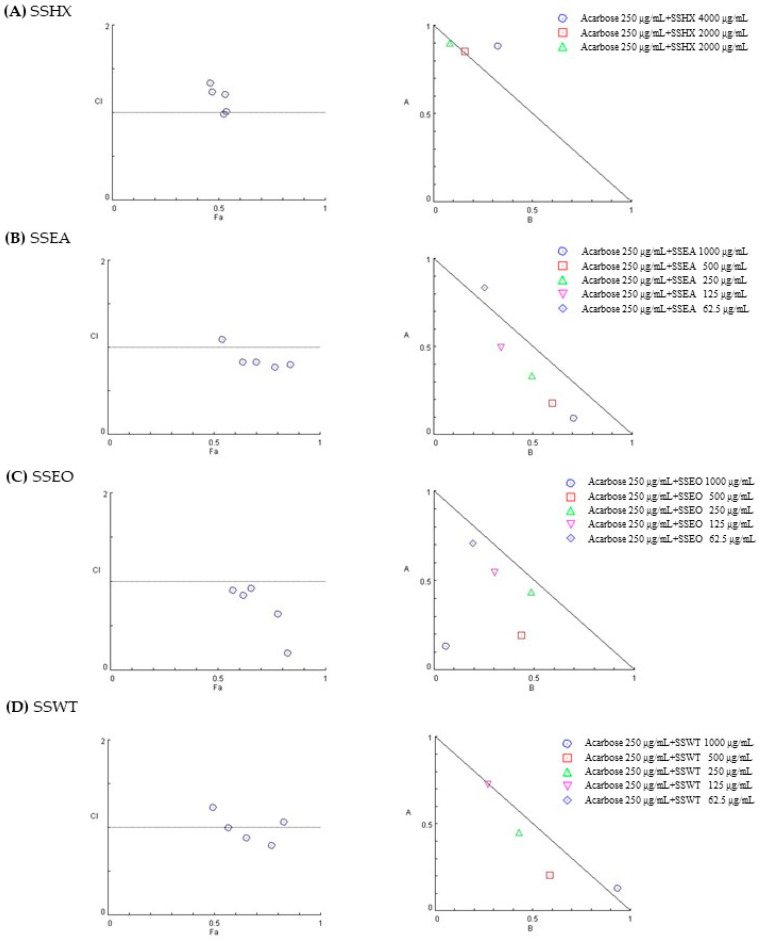
Fa-CI plots of SSHX, SSEA, SSEO, and SSWT, respectively (**A**–**D**). The normalized isobolograms of each extract are on the right.

In order to investigate the combined effects of *S. stramonifolium* extracts and a standard drug against alpha-glucosidase, five different extract concentrations covering their IC_50_ were combined with acarbose at 250 μg/mL. The plots of fraction-affected (Fa) versus combination index (CI) and the normalized isobologram of the combinations were generated as shown in Figure 1. If the CI values of the combination was lower than 1, it was indicated the synergistic effect of the combination. The normalized isobologram was used for the non-constant ratio combination. This normalized isobologram was constructed from the normalized dose of acarbose as the y-axis (A), and the normalized dose of extracts as the x-axis (B). The CI values of SSEO (62.5–1000 μg/mL), SSEA (125–1000 μg/mL), and SSWT (250–500 μg/mL) combined with acarbose exhibited synergistic effects. The combination of SSEO at 1000 μg/mL and standard acarbose showed a strong synergism effect, CI = 0.19. 

### 2.3. Isolation and Identification of Isolated Compounds

**Figure 2:**
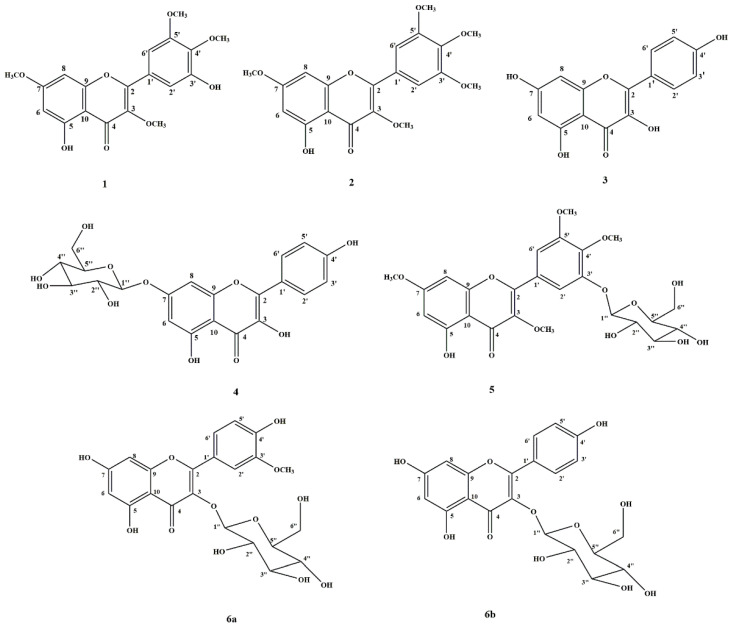
The isolated compounds from *S. stramonifolium* inflorescence.

The isolated compounds from *S. stramonifolium* inflorescence obtained five pure compounds and one mixed compound. Four of five pure compounds were known as myricetin 3, 4′, 5′, 7-tetramethyl ether (**1**); combretol (**2**); kaempferol (**3**); and kaempferol 7-*O*-*β*-glucopyranoside (**4**). However, another pure compound, 5-hydroxy 3-7-4′-5′-tetramethoxyflavone-3′-*O*-glucopyranoside (**5**), has not been reported in previous reviews. The mixed compound was the mixture of isorhamnetin 3-*O*-glucopyranoside (**6a**) and astragalin (**6b**). The chemical structures of *S. stramonifolium* isolated compounds are shown in Figure 2.

#### 2.3.1. Myricetin 3, 4′, 5′, 7-Tetramethyl ether (**1**)

Compound **1** was obtained as a yellow amorphous solid and dissolved in chloroform. The UV spectrum in chloroform showed an absorption maximum (λ_max_) at 270 and 330 nm. The IR spectrum demonstrated absorption bands at 3436, 2963, 1651, 1594–1455, 1260–1160, 1101–1027, and 803 cm^−1^. The ^1^H-NMR spectrum of **1** in CDCl_3_ exhibited four doublet and six singlet proton signals. Two of the doublet signals at δ_H_ 6.34 (1H, d, *J* = 2.19 Hz, H-6) and 6.42 (1H, d, *J* = 2.19 Hz, H-8) belong to *meta*-protons of ring A. Two other doublet signals at δ_H_ 7.34 (1H, d, *J* = 2.00 Hz, H-2′) and 7.33 (1H, d, *J* = 2.00 Hz, H-6′) were assigned *meta*-protons of ring B. The four of six singlet signals at δ_H_ 3.859 (3H, s, OCH_3_-7), 3.860 (3H, s, OCH_3_-3), 3.92 (3H, s, OCH_3_-5′), and 3.98 (3H, s, OCH_3_-4′) were determined as methoxyl protons, while two singlet signals at δ_H_ 5.90 (1H, brs, OH-3′) and 12.55 (1H, s, OH-5) were assigned as hydroxyl protons. The ^13^C-NMR spectrum showed nineteen carbon signals that were observed: eight oxygenated aromatic carbon signals at δ_C_ 137.74 (C-4′), 139.50 (C-3), 149.17 (C-3′), 152.03 (C-5′), 155.33 (C-2), 156.74 (C-9), 161.98 (C-5), and 165.57 (C-7); four tertiary aromatic carbon signals at δ_C_ 92.17 (C-8), 97.95 (C-6), 104.99 (C-6′), and 108.55 (C-2′); four methoxyl signals at δ_C_ 55.83 (OCH_3_-7), 56.07 (OCH_3_-5′), 60.33 (OCH_3_-3), and 61.11 (OCH_3_-4′); two quaternary carbon signals at δ_C_ 105.00 (C-10) and 125.95 (C-1′); and one carbonyl signal at δ_C_ 178.83 (C-4). The HMBC correlation suggested the structure substitution of **1**. The HR-ESIMS [M−H]^−^ ion peak at *m/z* 373.0929, correlated with a molecular formula of C_19_H_18_O_4_. The structure of **1** was determined to be myricetin 3,4′,5′,7-tetramethyl ether.

#### 2.3.2. Combretol (**2**)

Compound **2** was obtained as a yellow amorphous solid and dissolved in chloroform. The UV spectrum in chloroform showed an absorption maximum (λ_max_) at 268 and 347 nm. The IR spectrum demonstrated absorption bands at 3436, 2922–2850, 1657, 1601–1353, 1247–1126, 1048–1017, and 812–768 cm^−1^. The ^1^H-NMR spectrum of **2** in CDCl_3_ showed a singlet signal at δ_H_ 7.34 (2H, s, H-2′, and H-6′). Four other singlet signals at δ_H_ 3.854 (3H, s, OCH_3_-3), 3.860 (3H, s, OCH_3_-7), 3.926 (6H, s, OCH_3_-3′ and OCH_3_-5′), and 3.927 (3H, s, OCH_3_-4′) were determined as five methoxyl protons. The two doublets at δ_H_ 6.35 (1H, d, *J* = 2.19 Hz, H-6) and 6.45 (1H, d, *J* = 2.44 Hz, H-8) were assigned *meta*-protons of ring A. The signal at δ_H_ 12.57 (1H, s, OH-5) was the hydroxyl proton. The ^13^C-NMR spectrum displayed twenty carbon signals that were observed: eight oxygenated aromatic carbon signals at δ_C_ 139.39 (C-3), 140.50 (C-4′), 153.10 (C-3′ and C-5′), 155.61 (C-2), 156.70 (C-9), 162.03 (C-5), 165.50 (C-7), and 178.76 (C-4); four methoxyl carbon signals at δ_C_ 55.85 (OCH_3_-7), 56.31 (OCH_3_-3′ and OCH_3_-5′), 60.36 (OCH_3_-3), and 61.02 (OCH_3_-4′); four tertiary aromatic carbon signals at δ_C_ 92.26 (C-8), 97.92 (C-6), 105.90 (C-2′ and C-6′); and two quaternary carbon signals at δ_C_ 106.06 (C-10) and 125.45 (C-1′). The HMBC correlation suggested the structure substitution of **2**. The HR-ESIMS [M−H]^−^ ion peak at *m/z* 387.1086, correlated with a molecular formula of C_20_H_20_O_8_. The structure of **2** was determined to be 5-hydroxy-3,7,3′,4′,5′-pentamethoxyflavone or combretol.

#### 2.3.3. Kaempferol (**3**)

Compound **3** was obtained as a pale yellow amorphous solid and dissolved in methanol. The UV spectrum in methanol showed an absorption maximum (λ_max_) at 211, 265, and 366 nm. The ^1^H-NMR spectrum of **3** in CD_3_OD showed two doublet signals at δ_H_ 6.20 (1H, d, *J* = 2.00 Hz, C-6) and 6.42 (1H, d, *J* = 2.00 Hz, C-8) assigned as *meta*-protons of ring A. The other two doublet signals at δ_H_ 6.93 (2H, d, *J* = 8.50 Hz, C-3′, and C-5′) and 8.11 (2H, d, *J* = 8.50 Hz, C-2′ and C-6′) determined as the 1,4-disubstituted aromatic protons of ring B. The HR-ESIMS [M–H]^−^ ion peak at *m/z* 285.0407, correlated with a molecular formula of C_15_H_10_O_6_. The structure of **3** was determined to be kaempferol.

#### 2.3.4. Kaempferol 7-*O*-Glucopyranoside (**4**)

Compound **4** was obtained as a yellow amorphous solid and dissolved in methanol. The UV spectrum in methanol showed an absorption maximum (λ_max_) at 209, 254, and 363 nm. The ^1^H-NMR spectrum of **4** in DMSO-*d*_6_ exhibited the signals of typical kaempferol aglycone: four doublet signals at δ_H_ 6.42 (1H, d, *J* = 1.5 Hz, H-6), 6.80 (1H, d, *J* = 2.00 Hz, H-8), 6.94 (2H, d, *J* = 9.00 Hz, H-3′ and H-5′), and 8.09 (2H, d, *J* = 7.50 Hz, H-2′ and H-6′); a singlet signal at δ_H_ 12.53 (1H, s, OH-5). Moreover, the anomeric proton signal displayed at δ_H_ 5.07 (1H, d, *J* = 7.50 Hz, H-1″) and additional sugar signals appeared at δ_H_ 3.14–3.73 (m). The ^13^C-NMR spectrum showed nineteen carbon signals; however, they were indicated as twenty-one carbons: six oxygenated aromatic carbon signals at δ_C_ 136.72 (C-3), 147.96 (C-2), 156.20 (C-9), 159.84 (C-7), 160.82 (C-4′), and 163.13 (C-5); four tertiary aromatic carbon signals at δ_C_ 99.20 (C-8), 100.33 (C-6), 115.94 (C-3′ and C-5′) and 130.08 (C-2′ and C-6′); two quaternary aromatic carbons signals at δ_C_ 105.16 (C-10) and 122.05 (C-1′); carbonyl carbon signal at δ_C_ 176.09 (C-4); six carbon signals of hexosyl moiety at δ_C_ 61.07 (C-6″), 70.02 (C-4″), 73.59 (C-2″), 76.90 (C-3″), 77.64 (C-5″), and 94.81 (C-1″). The NOESY data of **4** showed the correlation signals between anomeric proton (H-1″) and two aromatic protons (H-6 and H-8). The HR-ESIMS [M–H]^−^ ion peak at *m/z* 447.0933, correlated with a molecular formula of C_21_H_20_O_11_. The structure of **4** was determined to be kaempferol-7-*O*-glucopyranoside.

#### 2.3.5. 5-Hydroxy 3-7-4′-5′-Tetramethoxyflavone-3′-*O*-glucopyranoside (**5**)

**Figure 3:**
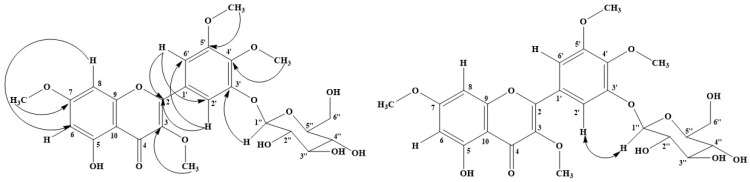
HMBC (on the left) and NOESY (on the right) correlations of compound **5**.

Compound **5** was obtained as a pale yellow amorphous solid and dissolved in methanol. The UV spectrum in methanol showed an absorption maximum (λ_max_) at 212, 268 and 337 nm. The ^1^H-NMR spectrum of **5** in DMSO-*d_6_* displayed the signals of typical flavonoid aglycone: two doublet signals at δ_H_ 6.41 (1H, d, *J* = 2.0 Hz, H-6) and 6.85 (1H, d, *J* = 2.50 Hz, H-8), and a singlet signal at δ_H_ 12.55 (1H, s, OH-5). Two other doublet signals at δ_H_ 7.46 (1H, d, *J* = 2.0 Hz, H-6′) and 7.52 (1H, d, *J* = 2.0 Hz, H-2′) belong to 1, 3, 4, 5-tetrasubstituted aromatic ring, ring B. Four singlet signals at δ_H_ 3.84 (3H, s, OCH_3_-4′), 3.87 (3H, s, OCH_3_-3), 3.88 (3H, s, OCH_3_-7), and 3.884 (3H, s, OCH_3_-5′) were determined as four methoxyl substitutions. Moreover, the anomeric proton signal exhibited at δ_H_ 4.96 (1H, d, *J* = 7.50 Hz, H-1″) and additional sugar signals appeared at δ_H_ 3.18–3.74 (m). The ^13^C-NMR spectrum demonstrated twenty-five carbon signals that were observed: eight oxygenated aromatic carbon signals at δ_C_ 139.30 (C-3), 141.25 (C-4′), 151.49 (C-3′), 153.28 (C-5′), 155.47 (C-2), 156.50 (C-9), 161.34 (C-5), and 165.80 (C-7); four tertiary aromatic carbon signals at δ_C_ 93.17 (C-8), 98.39 (C-6), 107.23 (C-6′), and 110.23 (C-2′); four methoxyl carbon signals at δ_C_ 56.59 (OCH_3_-5′), 56.61 (OCH_3_-7), 60.52 (OCH_3_-3), and 61.03 (OCH_3_-4′); two quaternary aromatic carbons signals at δ_C_ 105.81 (C-10) and 122.05 (C-1′); carbonyl carbon signal at δ_C_ 178.71 (C-4); and six carbon signals of hexosyl moiety at δ_C_ 61.34 (C-6″), 70.27 (C-4″), 73.76 (C-3″), 77.22 (C-2″), 77.89 (C-5″), and 101.92 (C-1″). Its 1D-NMR data resembled NMR data of **1**. The obvious difference was the C-4′ chemical shift of **5,** which was downfield than **1**. The data were also compared with 3′-*O*-*β*-D-(4″-*O*-methylglucopyranosylo)-5, 7, 4′, 5′-tetramethoxylavonone [10], except for the presence of sugar at C-3′. The HMBC correlation from H-1″ to C-3′ (Figure 3) indicated glucose substitution. Moreover, NOESY cross peak exhibited space correlation between H-2′ and H-1″ as Figure 3. The HR-ESIMS [M–H]^−^ ion peak at *m/z* 535.1457, correlated with a molecular formula of C_25_H_28_O_13_. Therefore, the structure of **5** was determined as 5-hydroxy 3-7-4′-5′-tetramethoxyflavone-3′-*O*-glucopyranoside.

#### 2.3.6. Mixture Compound (**6**)

Mixture compound **6** was obtained as a yellow amorphous solid and dissolved in methanol. The UV spectrum in methanol showed an absorption maximum (λ_max_) at 260, 292, and 370 nm. The high-performance liquid chromatography (HPLC) with hypersil ODS column (250 × 4.0 mm, 5 μm particle size) and diode array detector exhibited two peaks of **6a** and **6b** (see Supplementary Materials, Figure S1).

##### Isorhamnetin 3-*O*-Glucopyranoside (**6a**)

The ^1^H-NMR spectrum of **6a** DMSO-*d_6_* displayed the signals of substitution patterns of aromatic rings (ring A and ring B) of flavonoid. Two doublet signals at δ_H_ 6.18 (1H, m, H-6), 6.40 (1H, m, H-8), and a singlet signal at δ_H_ 12.60 (1H, s, OH-5) indicated the presence of 5, 7-dihydroxy aromatic ring, ring A. Two other doublet signals at δ_H_ 6.91 (1H, d, *J* = 8.5 Hz, H-5′) and 7.95 (1H, d, *J* = 2.1 Hz, H-2′) and a doublet of a doublet at δ_H_ 7.48 (1H, dd, *J* = 2.1 and 8.5 Hz, H-6′) presented 1,3,4-trisubstituted aromatic ring, ring B. The anomeric proton signal exhibited at δ_H_ 5.58 (1H, d, *J* = 7.0 Hz, H-1″) and other sugar signals appeared at δ_H_ 3.11–3.84 (m). Moreover, the methoxyl signal was found at δ_H_ 3.84 (3H, s, OCH_3_-3′). The ^13^C-NMR spectrum demonstrated twenty-two carbon signals that were observed: seven oxygenated aromatic carbon signals at δ_C_ 133.57 (C-3), 147.34 (C-3′), 149.86 (C-4′), 156.52 (C-2), 156.95 (C-9), 161.65 (C-5), and 165.80 (C-7); five tertiary aromatic carbon signals at δ_C_ 94.31 (C-8), 99.48 (C-6), 113.91 (C-2′), 115.66 (C-5′), and 122.44 (C-6′); two quaternary aromatic carbons signals at δ_C_ 104.07 (C-10) and 121.56 (C-1′); carbonyl carbon signal at δ_C_ 177.75 (C-4); methoxyl carbon signals at δ_C_ 56.11 (OCH_3_-5′), six carbon signals of hexosyl moiety at δ_C_ 61.29 (C-6″), 70.34 (C-4″), 74.69 (C-3″), 76.88 (C-2″), 77.96 (C-5″), and 101.38 (C-1″). The HMBC correlation from OCH_3_-5′ to C-3′ indicated methoxyl substitution. The data were also compared with 3′-*O*-methylquercetin-3-*O*-glucopyranoside [11]. The negative HR-ESIMS [M−H]^−^ ion peak at *m/z* 477.1042 correlated with a molecular formula of [(C_22_H_22_O_12_)-H]^−^. Thus, the structure of **6a** was determined as 3′-*O*-methylquercetin-3-*O*-glucopyranoside or isorhamnetin 3-*O*-glucopyranoside.

##### Astragalin (**6b**)

The ^1^H-NMR spectrum of **6b** DMSO-*d_6_* exhibited a signal pattern of 5, 7-dihydroxy aromatic ring, ring A. Two doublet signals at δ_H_ 6.18 (1H, m, H-6), 6.40 (1H, m, H-8), and a singlet signal at δ_H_ 12.60 (1H, s, OH-5) were observed. It also had a doublet signal at 6.88 (2H, d, *J* = 8.5 Hz, H-3′ and H-5′) and a doublet of doublet signal at 8.04 (2H, dd, *J* = 8.5 and 2.1 Hz, H-2′ and H-6′). These suggested that the ring B of **6b** was the 1,4-disubstituted aromatic ring. The anomeric proton signal at δ_H_ 5.45 (1H, d, *J* = 7.5 Hz, H-1″) and other sugar signals appeared at δ_H_ 3.03–3.23 (m) indicating the presence of a hexose moiety. The ^13^C-NMR spectrum demonstrated nineteen carbon signals that were observed: six oxygenated aromatic carbon signals at δ_C_ 133.33 (C-3), 156.50 (C-2), 156.95 (C-9), 160.42 (C-4′), 161.65 (C-5), and 165.80 (C-7); four tertiary aromatic carbon signals at δ_C_ 94.31 (C-8), 99.48 (C-6), 115.66 (C-3′ and C-5′), and 131.32 (C-2′ and C-6′); two quaternary aromatic carbon signals at δ_C_ 104.07 (C-10) and 121.38 (C-1′); carbonyl carbon signal at δ_C_ 177.69 (C-4); six carbon signals of hexosyl moiety at δ_C_ 61.03 (C-6″), 70.26 (C-4″), 74.80 (C-3″), 76.71(C-2″), 77.90 (C-5″), and 101.27 (C-1″). The HMBC and COSY correlation suggested the structure substitution of **6b**. The negative HR-ESIMS [M−H]^−^ ion peak at *m/z* 447.0938 correlated with a molecular formula of [(C_21_H_20_O_11_)-H]^−^. Therefore, the structure of **6b** was defined as kaempferol 3-*O*-glucopyranoside or astragalin.

### 2.4. Alpha-Glucosidase Inhibition Assay

Five pure compounds and a mixture compound were obtained with trace amount. Thus, their alpha-glucosidase inhibitory activities were analyzed as the percentages of inhibition at 200 or 400 μg/mL (as presented in Table 2). They exhibited weaker alpha-glucosidase inhibition than the positive control, acarbose.

### 2.5. Enzyme Kinetic Assay

**Figure 4:**
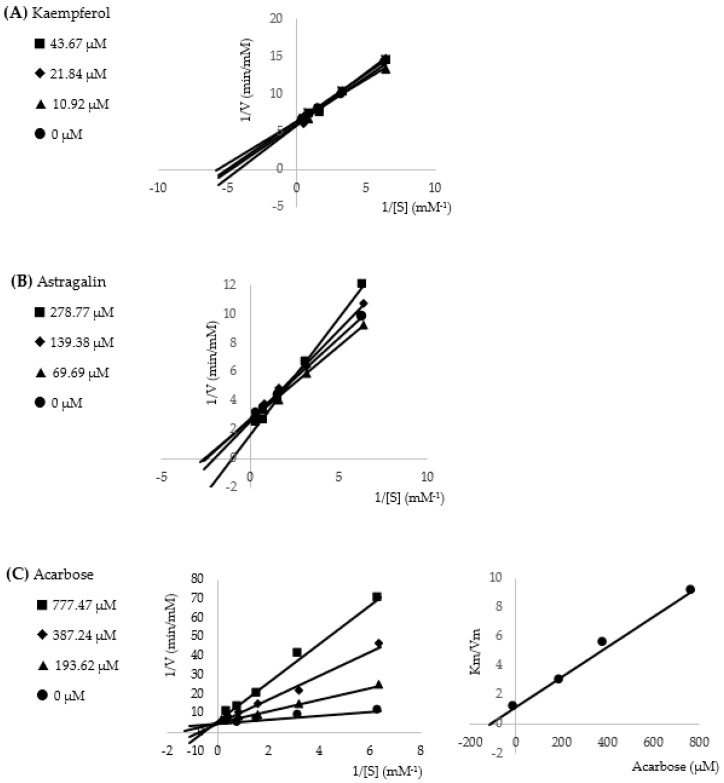
Lineweaver−Burk plots of kaempferol (**A**), astragalin (**B**). and acarbose (**C**). The secondary plot of acarbose is on the right.

To investigate the IC_50_ and type of inhibition of flavonoid compounds, the standard kaempferol and astragalin were purchased and used as the representatives. In the tested condition, due to the solubility problem of flavonoid compounds, DMSO was used as the co-solvent with the limitation that it was not more than 5%. The IC_50_ values of kaempferol, astragalin, and acarbose are shown in Table 3. Both of the representative flavonoids exhibited less alpha-glucosidase inhibitory activity than the acarbose standard. The double reciprocal Lineweaver–Burk plot of kaempferol and astragalin was used to perform mixed-type inhibition as shown in Figure 4, while acarbose showed a competitive inhibition with the inhibition constant (Ki) value as 110.48 μM.

### 2.6. Molecular Docking

**Figure 5:**
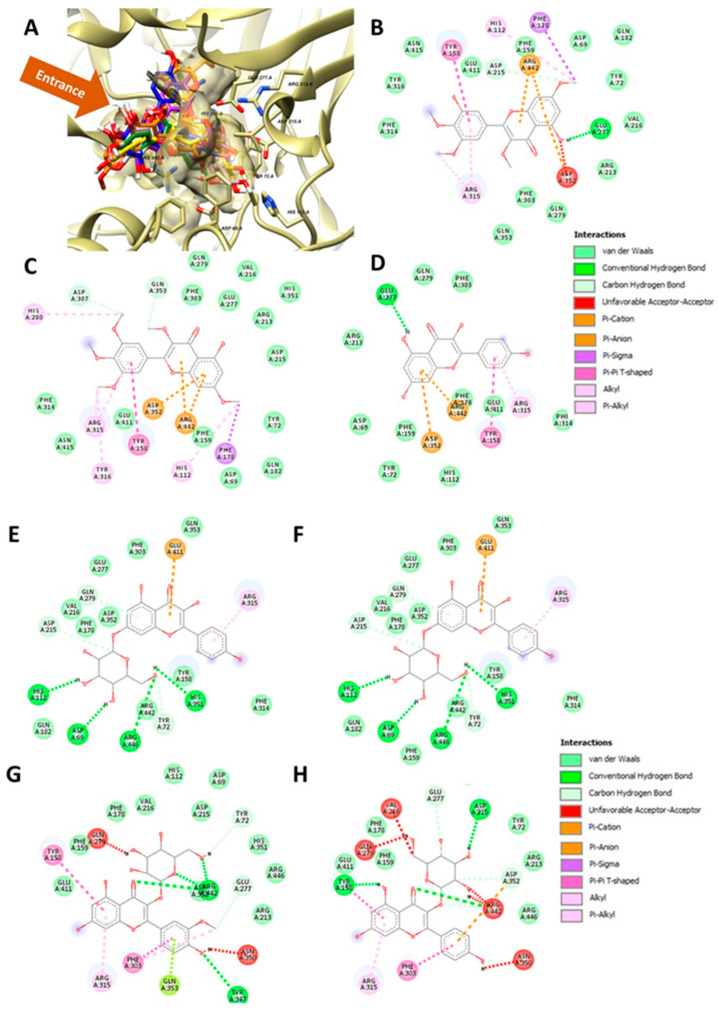
Molecular interactions of the obtained flavonoids from *S. stramonifolium* and alpha-glucosidase. (**A**) Three-dimensional diagram of all docked flavonoid molecules at the entrance of the active site of alpha-glucosidase. (**B**–**H**) Two-dimensional diagrams of molecular interactions of myricetin 3, 4′, 5′, 7-tetramethyl ether (compound **1**, (**B**)), combretol (compound **2**, (**C**)), 5-hydroxy 3-7-4′-5′-tetramethoxyflavone-3′-*O*-glucopyranoside (compound **5**, (**D**)), kaempferol (compound **3**, (**E**)), kaempferol-7-*O*-glucopyranoside (compound **4**, (**F**)), isorhamnetin-3-*O*-glucopyranoside (compound **6a**, (**G**)), and astragalin (compound **6b**, (**H**)), and amino acid residues at the catalytic site of alpha-glucosidase.

After alpha-glucosidase inhibitory evaluation, we performed molecular docking to observe possible interactions between the obtained flavonoids and the alpha-glucosidase enzyme. First, we divided our flavonoids into three sub-groups: myricetin derivatives, kaempferol derivatives, and others. Our docking showed that all sub-groups were laid on the same site, blocking the substrate from entering the active site (Figure 5A). Furthermore, we provided molecular interactions between each obtained flavonoid and amino acid residues in the active site of alpha-glucosidase in Figure 5B–H. Noticeably, at least five amino acid residues, TYR158, GLU277, ARG315, ASP352, and ARG442, were highly conserved in all flavonoids obtained from *S. stramonifolium*, as shown in Table 4. On the other hand, we observed that some residues were distinctively conserved in a specific sub-group; for example, TYR72 was only conserved among the kaempferol derivatives, while HIS112, PHE178, HIS280, an d TYR316 were conserved among the myricetin derivatives. We have provided a structural alignment of each flavonoid sub-group in the supplementary materials; see Figures S2 and S3.

We further rescored the binding energy using Autodock4.2.6 to evaluate more energetic parameters (Table 5). As a result, we found a contradiction in the obtained binding energies between two docking programs between compound **1** and **2** from the myricetin derivatives sub-group, while the others were in agreement, as shown in Table 5. It is worth noting that each applied docking program used a different calculation approach (scoring function and searching algorithm) [12]. Therefore, it is possible to obtain contradictory results between these two programs. However, comparing the docking result with the experimental data is essential. Thus, we combined the results from both docking and in vitro experiments. We found that a rescored binging energy of compounds **1** and **2** from Autodock 4.2.6 disagreed with Autodock Vina and an in vitro alpha-glucosidase inhibitory activity (Table 5). As a result, we did not further evaluate the obtained energic parameters of these two compounds. On the other hand, the binding energies of compounds **2** and compound **5** from both docking programs agreed with an in vitro experiment showing less inhibitory activity (more binding energy) from a glycosylated compound than a non-glycosylated one. Noticeably, we found numerous higher energy levels from Van der Waals (vdW) and hydrogen bondings (Hbond), indicating less potency in enzyme inhibition from compound **5** than compound **2**. Moreover, we also observed that desolvation and torsion-free energies were slightly higher in the glycosylated compound. Interestingly, we found a similar trend between compounds **3** and **6b**, demonstrating a greater vdW and Hbond energies. However, compound **4** demonstrated a smaller vdW and Hbond energies than compounds **3** even though compound **4** also presented with the same glucose moiety as compound **6b**. Therefore, we found that a sugar position on the flavonoid structure affected alpha-glucosidase inhibition. Finally, we observed that compound **6a** presented with O-glycosylation at the C-3 position, showing a smaller vdW and Hbond energies, unlike compound **6b**. However, both compounds **6a** and **6b** shared the same glycosylation position (C-3). Therefore, based on our findings here, not only a sugar position but also the flavonoids class is responsible for alpha-glucosidase inhibitory activity.

## 3. Discussion

According to the results, the extract of *S. stramonifolium* inflorescence showed strong inhibition with IC_50_ 81.27 μg/mL over the acarbose standard, IC_50_ 241.40 μg/mL. Therefore, *S. stramonifolium* inflorescence was selected for this study. The extraction used the step-up solvent polarity extraction from *n*-hexane, EtOAc, ETOH and boiled water, respectively. Among these extracts, the ethyl acetate extract (SSEA) and ethanol extract (SSEO) showed high inhibitory activity with IC_50_ 215.92 and 221.67 μg/mL, respectively. Some reviews have suggested that one of the major compounds from the *Solanum* species was flavonoids [9]. Therefore, the TPC and TFC of all extracts were also determined. The maximum levels of TPC and TFC were observed in SSEO as 0.261 ± 0.003 mg GEA/g extract and 69.012 ± 2.457 mg CAE/g extract, respectively. These related results of the alpha-glucosidase inhibition, TPC and TFC, may be noted that the flavonoid compounds are one of the contributors to the glucosidase inhibition of *S. stramonifolium*. To investigate the combined effect of the standard acarbose and *S. stramonifolium* extracts against alpha-glucosidase, the in vitro experiment was performed as a non-constant ratio combination. From the normalized isobologram, the normalized doses of SSEA (125–1000 μg/mL), SSEO (62.5–250 μg/mL) and SSWT (250–500 μg/mL), which, combined with acarbose, were increased, which relates to the increase in the extract concentrations. These facts may imply that the increasing of SSEA, SSEO, and SSWT concentrations enhanced the ability of acarbose to inhibit alpha-glucosidase activity. Interestingly, the normalized dose plots of SSEO at 500 and 1000 μg/mL combined with acarbose at 250 μg/mL were close to the right angle. This may indicate that the inhibition of these two combinations resulted from strong synergism. Furthermore, the compounds of *S. stramonifolium* were isolated by using chromatographic techniques, and their chemical structures were determined using spectroscopic techniques, especially NMR and HRMS analysis. The isolated compounds were three flavonoids (compound **1**–**3**), two flavonoid-glycosides (compound **4** and **5**), and one mixture of flavonoid and glycoside (compound **6**). The presence of *O*-glycosylation at C-3′ position of compound **5** has not been reported before. Therefore, this was the first report of compound **5**. 

Due to the low yield isolation, each compound could be only evaluated for the percentage of alpha-glucosidase inhibition, which was not enough to further determine the IC_50_ value. Therefore, two flavonoid standards, kaempferol and astragalin, were purchased and used as the representatives for the mode of action analysis. The kinetic prediction showed that kaempferol and astragalin inhibited the activity of alpha-glucosidase enzyme in a mixed manner, while the acarbose exhibited it in competitive manner. Kaempferol has been determined as both a mixed-type inhibitor [13,14] and a non-competitive inhibitor [15], while astragalin has been reported as a non-competitive inhibitor [16]. It is known that the competitive manner is the inhibitor bond free enzyme (E) at the active site, the uncompetitive manner is the inhibitor bond with the enzyme-substrate (ES) complex, the non-competitive manner is the inhibitor bond with the E and ES complex, and the mixed-type manner is the inhibitor bond target enzyme at more than one site [17]. The different manner of enzyme inhibition may result from the different conditions tested.

For a sophisticated understanding, molecular docking was performed, and the link between glycosylation and its attached position on the flavonoid structures was evaluated to compare their intermolecular binding parameters. Although the structure–activity relationship (SAR) of flavonoids and alpha-glucosidase has been intensively studied, only limited studies have investigated an impact of glycosylation and its position on a flavonoid structure in terms of binding energy [18,19,20]. We know that *O*-glycosylation at C-3 and C-7 of flavonoid structure weakens the anti-alpha-glucosidase activity [18]. However, the reason is still unclear. Our molecular docking showed that 3-*O*-glycosylation from compound **6b** caused a much higher vdW and Hbond energy, indicating lower stability in molecular binding than a non-glycosylated one. However, this is not true in the case of compound **6a**, showing lower vdW and Hbond energies from the same C-3-*O*-glycosylation. Therefore, we found that not only 3-*O*-glycosylation but also flavonoid structures altered the binding energy through vdW and Hbond energies, affecting alpha–glucosidase inhibitory activity.

7-*O*-glycosylation from compound **4** exhibited noticeably lower vdW and Hbond energies than non-glycosylated compound **3**. However, considering only vdW and Hbond energies was not enough to explain the in vitro result. Therefore, we evaluated the other parameters and found that desolvation energy compensated for a lower energy level of vdW and Hbond energies, resulting in higher binding energy, which indicates a lower inhibition. As a result, we observed that glycosylation affects both vdW and Hbond energy and desolvation energy. Therefore, we proposed that these two parameters were essential for glycosylated flavonoids’ alpha-glucosidase inhibition. Furthermore, we could identify high conserved amino acid residues such as TYR158, GLU277, ARG315, ASP352, and ARG442 at the catalytic domain of alpha-glucosidase [21,22] among all isolated flavonoids from *S. stamonifolium*. Therefore, these conserved amino acid residues can be used as a potential target for further designing more potent flavonoid derivatives.

## 4. Experimental Section

### 4.1. General

The phytochemical isolation was performed using chromatographic techniques with classical column chromatography of silica gel 60 (Merck, Darmstadt, Germany), LiChroperp^®^ RP-18 (Merck, Darmstadt, Germany), and Sephadex^®^ LH-20 (Bio-Sciences, Uppsala, Sweden). The purified investigation of isolated compounds was performed by thin-layer chromatography (TLC) plate pre-coated with silica gel 60 F_254_ (Merck, Darmstadt, Germany) or by high-performance liquid chromatography (HPLC) analysis (Algilant Technologies, Waldbronn, Germany). Fourier Transform NMR Spectrometer 500 MHz (Varian, Frankfurt, Germany) was used to create one-dimensional and two-dimensional NMR spectra by using dimethyl sulfoxide (DMSO-*d*_6_), chloroform (CDCl_3_), and methanol (CD_3_OD) for dissolving the isolated compounds. UV-Spectrophotometer Genesis 6 (Thermo, Frankfurt, Germany) was used for the measurement of UV/Vis spectra of the isolated compounds. IR spectra of each isolated compound were reported from Spectrum One FT-IR Spectrometer (PerkinElmer, Buckinghamshire, UK). Liquid chromatograph-quadrupole time of fight mass spectrometer (Agilent, Santa Clara, CA, USA) was used for the analysis of high-resolution electron spray ionization mass spectrometry (HRESIMS). The isolated compounds (myricetin 3,4′,5′,7-tetramethyl ether (**1**), combretol (**2**), kaempferol (**3**), kaempferol 7-*O*-glucopyranoside (**4**), and 5-hydroxy 3-7-4′-5′-tetramethoxyflavone-3′-*O*-glucopyranoside (**5**)) have purity higher than 95%.

### 4.2. Plant Material

The inflorescence of *S. stamonifolium* was collected in 2014–2015 from Songkhla province, Thailand, and identified by the botanist, Ms. Ramrada Meeboonya. The voucher specimens were deposited at Forest Herbarium, Department of National Parks, Thailand. The reference numbers are BKF No. 189326 and BKF No. 189327.

### 4.3. Extraction and Isolation

The dried inflorescence of *S. stamonifolium* (487.61 g) was macerated with *n*-hexane at room temperature for 3 days in triplicate. The extracted *n*-hexane solvent was evaporated to remove the solvent and yield the crude extract, named SSHX (11.95 g). Next, the marc was re-macerated by ethyl acetate and ethanol, respectively, to obtain SSEA (4.10 g) and SSEO (7.34 g). Finally, the marc was boiled with filtrated water to yield SSWT (144.10 g).

The SSEA was subjected to a silica column by using the gradient mobile phase of CHCl_3_ to CHCl_3_-MeOH (1:1) to give 37 fractions (SSEA_A1–A37_). The fractions SSEA_A24–A32_ were the amorphous substances. They were pooled and washed with MeOH. Then, they were loaded on TLC by using the mixed solvent of *n*-hexane-EtOAc-CHCl_3_ (6:2:2) to produce compound **1** (6.0 mg).

The SSEO was loaded onto silica flash column using the gradient of *n*-hexane to EtOAc-MeOH (1:1) to achieve 45 fractions (SSEO_A1–A45_). The fraction SSEO_A12_ was separated by Sephadex^®^ LH-20 column to obtain compound **1** (1.0 mg).

The SSHX was chromatographed on a silica column by using the isocratic solvent of *n*-hexane-EtOAc (9:1) to 60 fractions (SSHX_A1–A60_). Fractions SSHX_A32–A37_ were pooled and subjected to a silica column and using gradient mobile phase of *n*-hexane to *n*-hexane-EtOAc (7:3) to achieve 127 fractions (SSEO_B1–B127_). The fractions SSEO_B18–B25_ were combined and separated by silica column with gradient mobile phase of *n*-hexane to EtOAc -MeOH (9:1) to give 186 fractions (SSEO_C1–C186_). Fractions SSEO_C47-C176_ were pooled and loaded on TLC by using the mixed solvent of *n*-hexane-EtOAc-CHCl_3_ (6:2:2) to produce compound **2** (3.1 mg). Moreover, the fractions SSEO_B39–B49_ were combined and chromatographed on silica column by using the isocratic solvent of *n*-hexane-EtOAc-CHCl_3_ (6:2:2) to 38 fractions (SSHX_D1–D60_). Fractions SSHX_D14–D34_ were combined and separated by Sephadex^®^ LH-20 column using CHCl_3_-MeOH (2:8) to obtain compound **1** (2.0 mg).

The SSWT was separated by liquid–liquid extraction to receive 4 fractions, *n*-hexane-SSWT, CHCl_3_-SSWT, EtOAc-SSWT, and water-SSWT. The EtOAc-SSWT was loaded to a silica flash column using the gradient of *n*-hexane to EtOAc-MeOH (7:3) to give 22 fractions (EtOAc-SSWT_A1–A22_). The fractions of EtOAc-SSWT _A10–A11_ were pooled and separated by Sephadex^®^ LH-20 column using MeOH to produce compound **3** (2.0 mg). On the other hand, fraction EtOAc-SSWT_A15_ was loaded to Sephadex^®^ LH-20 column using MeOH to obtain 63 fractions (EtOAc-SSWT_B1–B63_). The pooled fractions of EtOAc-SSWT _B58-B63_ were compound **4** (2.0 mg). The fractions of EtOAc-SSWT_B36–B39_ were combined and separated by Sephadex^®^ LH-20 column using MeOH to obtain 39 fractions (EtOAc-SSWT_C1–C39_). The fractions of EtOAc-SSWT_C20–C39_ were pooled and loaded on TLC by using the mixed solvent of CHCl_3_-MeOH (9:1) to produce compound **5** (1.0 mg). Another separation, the pooled fractions of EtOAc-SSWT_B48–B50_, were loaded to reverse column chromatography using the mixed solvent of MeOH-H_2_O (1:1) to obtain compound **6** (1.6 mg).

### 4.4. Enzymatic Assay

All extracts and isolated compounds were examined for their alpha-glucosidase inhibitory activity according to Dej-adisai and Pitakput, 2015 [23]. Briefly, 0.01 M phosphate buffer (50 μL), samples or standard solution (50 μL), and alpha-glucosidase enzyme solution (50 μL) were added to the well plate. The mixture solution was incubated at 37 °C for 2 min. Next, *para*-nitrophenyl-α-D-glucopyranoside solution (50 μL) was added. The enzymatic inhibition was evaluated from the yellow final product, *p*-nitrophenol (*p*NP). The color of the reaction was detected by visible light at 405 nm of SPECTROstar Nano spectrophotometer (BMG Labtech, Ortenberg, Germany). The product absorption was converted to velocity (V) by using Equation (1). Then, the velocity was used to calculated the percentage of inhibition by Equation (2). Furthermore, the ability of sample was performed by IC_50_ value, which was generated from the calibration curve of percentages of inhibition at five different sample concentrations.

Velocity = ∆ Absorbance at 405 nm/∆ Time(1)

% Inhibition = [(V_control_ − V_sample_)/V_control_] × 100 (2)

### 4.5. Total Phenolic Content (TPC)

In order to determine the TPC of extracts, the Folin–Cicocateu colorimetric assay was used according to Chanthasri et al. [22]. Briefly, each extract at a concentration of 2.5 mg/mL (120 μL) was mixed with 10-fold diluted Folin–Ciocalteu reagent (1 mL). Five minutes later, 20% *w/v* of sodium carbonate solution (1 mL) was added. The mixed solutions were left to rest for 90 min at room temperature. Then, they were measured for their absorbance at 725 nm (Sunrise^TM^, Männedorf, Germany). TPC was calculated from the calibration curve using the gallic acid standard. The results were reported as the mg of gallic acid equivalent/mg extract.

### 4.6. Total Flavonoid Content (TFC)

TFC of extracts was also determined by the aluminum chloride colorimetric assay following Chanthasri et al. [24]. Briefly, each extract at a concentration of 2.5 mg/mL (50 μL) was mixed with distilled water (1 mL), 5% *w/v* of sodium nitrite (300 μL) and 10% *w/v* of aluminum trichloride (300 μL). The mixed solution was left to rest for 6 min at room temperature. The 1 M sodium hydroxide (20 mL) was added and was adjusted to 10 mL by using distilled water. Ten minutes later, the solutions were measured for their absorbance at 510 nm (Sunrise^TM^, Männedorf, Germany). TFC was calculated from the calibration curve using the catechin standard. The results were reported as the mg of catechin equivalent/mg extract.

### 4.7. Combination Study

Compusyn Software from ComboSyn, Inc. (Paramus, NJ, USA) (http://www.combosyn.com/, accessed on 1 November 2021) was used to determine the combined effect of plant extracts and standard acarbose in this study. The Chou–Talay method for drug combination was described in the previous report [3]. The plot of fraction affected (Fa) versus combination index (CI) and normalized isobologram was received from automated computer simulation. CI < 1, CI = 1, and CI > 1 were defined synergism, additive effect, and antagonism, respectively.

### 4.8. Enzyme Kinetic Study

To determine the mode of inhibition, the enzyme kinetic procedure was performed as described by Sakulkeo et al. [25]. The double reciprocal Lineweaver–Burk plot was constructed and used for the mode of action determination. The inhibition constant (Ki) was conducted from the secondary plot. Briefly, the 5 concentrations of substrate *p*NPG (2.5–0.125 mM), the enzyme from *Saccharomyces cerevisieae* (EC 3.2.1.20) at 1 unit/mL, and 3 concentrations of sample were tested in triplicate.

### 4.9. Molecular Docking

The authors used AutoDock Vina version 1.1.2 and Autodock 4.2. (The Scripp Research Institute, San Diego, CA, USA) to proceed with the molecular interaction prediction [26,27,28]. Previous studies described the method used here in this study [25,28,29,30]. Briefly, the alpha-glucosidase from *Saccharomyces cerevisiae* (PDB ID: 3a4a) was downloaded from RCSB Protein Data Bank (https://www.rcsb.org/, accessed on 14 May 2022), while the compounds were downloaded from Pubchem (https://pubchem.ncbi.nlm.nih.gov/, accessed on 14 May 2022). The target protein was prepared, and Autodock Tool version 1.5.6 was used to identify the active site. To indicate the active site, the native glucose molecule was used as the navigator [31]. The active site was presented as the three-dimensional grid box (X-axis = 21.1, Y-axis = −7.4, and Z-axis = 24.2). The grid size was set as 16 Å × 16 Å × 16 Å. Moreover, the compounds of interest were downloaded from Pubchem (https://pubchem.ncbi.nlm.nih.gov/, accessed on 14 May 2022). Avogadro version 1.2.0 was used to optimize the geometry and general amber force field (GAFF) [32]. In the experiment, all parameters were set as default except the exhaustiveness was adjusted as 24. The re-docking of the native ligand was measured to validate the protocol. The RMSD of re-docking was 0.936 Å, which passed the acceptance criterion (RMSD < 1 Å). To analyze all results, the ViewDock package from Chimera version 1.11.2 (UCSF, San Diego, CA, USA) was used to visualize and evaluate the outcomes [33].

## 5. Conclusions

Six compounds were isolated from *S. stramonifolium* inflorescence, including a new flavonoid-glycoside (5-hydroxy 3-7-4′-5′-tetramethoxyflavone-3′-*O*-glucopyranoside), four known flavonoid derivatives (myricetin 3, 4′, 5′, 7-tetramethyl ether, combretol, kaempferol, and kaempferol 7-*O*-glucopyranoside), and one mixture of known flavonoid-glycoside (isorhamnetin 3-*O*-glucopyranoside and astragalin). The crude extracts were also studied on the anti-alpha-glucosidase activity. The combination of ethyl acetate, ethanol, and water extracts with acarbose exhibited a synergistic effect. Kaempferol and astragalin were chosen for a mechanism of action analysis. Both of them displayed a mixed-type inhibitory manner. The docking study provided a molecular understanding of the interaction between alpha-glucosidase enzyme and flavonoid compounds. The flavonoid class and a sugar position on the flavonoid structure affected the alpha-glucosidase inhibition. The results presented in this study provided scientific evidence for *S. stramonifolium* as an essential flavonoid resource and for designing more potent flavonoid derivatives to inhibit an alpha-glucosidase enzyme in the future.

## Figures and Tables

**Table 1:** Extraction yield, alpha-glucosidase inhibition, TPC, and TFC of *S. stramonifolium* crude extracts.

Extracts	% Yield Extraction	% Inhibition at 2 mg/mL	IC_50_ (µg/mL)	TPC mg GAE/g Extract	TFC mg CAE/g Extract
SSIE	6.35	97.98 ± 0.64	81.27	0.318 ± 0.001	80.541 ± 2.010
SSHX	2.45	11.65 ± 9.44	3.13 ^a^	0.034 ± 0.000	21.247 ± 0.623
SSEA	1.01	92.79 ± 1.07	215.92	0.073 ± 0.002	25.012 ± 0.471
SSEO	1.51	96.63 ± 0.65	221.67	0.261 ± 0.003	69.012 ± 2.457
SSWT	29.55	69.34 ± 1.76	324.44	0.118 ± 0.001	27.600 ± 0.471
Acarbose (Positive control)	-	76.44 ± 3.06	241.40	-	-

^a^ mg/mL; GAE: gallic acid equivalent, CAE: catechin equivalent.

**Table 2:** Inhibitory activity of isolated compounds from *S. stramonifolium* on alpha-glucosidase.

Compounds	% Inhibition	
	at 400 μg/mL	at 200 μg/mL
Myricetin 3, 4′, 5′, 7-tetramethyl ether (**1**)	12.78 ± 4.89	-
Combretol (**2**)	8.51 ± 3.79	-
Kaempferol (**3**)	13.01 ± 2.69	-
Kaempferol 7-*O*-glucopyranoside (**4**)	7.68 ± 0.33	-
5-Hydroxy 3-7-4′-5′-tetramethoxyflavone-3′-O-glucopyranoside (**5**)	-	0.84 ± 0.21
Mixture of isorhamnetin 3-*O*-glucopyranoside (**6a**) and astragalin (**6b**)	1.99 ± 5.42	-
Acarbose (Positive control)	60.78 ± 3.61	45.27 ± 4.5

**Table 3:** Alpha-glucosidase inhibitory activity of selected flavonoid compounds.

Compounds	IC_50_ (µg/mL)	Inhibition Type	Ki (µM)
Kaempferol	585.63	Mixed	-
Astragalin	>600	Mixed	-
Acarbose (Positive control)	191.12	Competitive	110.48

**Table 4:** Molecular interactions between amino acid residues from alpha-glucosidase and compounds of interest and conserved residues.

No	Residues	Compounds							Consensus
		Myricetin Derivatives			Kaempferol Derivatives			Other	
		1	2	5	3	4	6b	6a	
1	ASP69	-	-	-	-	**✓**	-	-	1
**2**	**TYR72**	**-**	**-**	**-**	**-**	**✓**	**✓**	**-**	**2**
**3**	**HIS112**	**✓**	**✓**	**-**	**-**	**✓**	**-**	**-**	**3**
**4**	**TYR158**	✓	✓	✓	✓	-	✓	✓	**6**
5	PHE159	-	-	✓	-	-	-	-	1
**6**	**PHE178**	**✓**	**✓**	**✓**	**-**	**-**	**-**	**-**	**3**
7	ASP215	✓	-	✓	-	✓	-	✓	4
8	VAL216	-	-	✓	-	-	-	✓	2
9	GLU277	**✓**	-	**✓**	**✓**	-	**✓**	**✓**	5
10	GLN279	-	-	-	-	-	✓	✓	2
**11**	**HIS280**	**-**	**✓**	**✓**	**-**	**-**	**-**	**-**	**2**
12	PHE303	-	-	-	-	-	✓	✓	2
13	THR306	-	-	✓	-	-	-	-	1
14	ASP307	-	✓	-	-	-	-	-	1
15	ARG315	✓	✓	✓	✓	✓	✓	✓	7
**16**	**TYR316**	**-**	**✓**	**✓**	**-**	**-**	**-**	**-**	**2**
17	TYR347	-	-	✓	-	-	✓	-	2
18	ASN350	-	-	✓	-	-	✓	✓	3
19	HIS351	-	-	-	-	✓	-	-	1
**20**	**ASP352**	✓	✓	✓	✓	-	✓	✓	**6**
21	GLN353	-	-	✓	-	-	✓	-	2
22	GLU411	-	-	-	-	✓	-	-	1
23	ASN415	-	-	✓	-	-	-	-	1
**24**	**ARG442**	✓	✓	✓	✓	-	✓	✓	**6**
25	ARG446	-	-	-	-	✓	-	-	1
	Total	8	9	16	5	8	11	10	

Highlight indicates conserved residues in all sub-groups, while bold indicates conserved residues in a particular sub-group.

**Table 5:** Obtained binding energy of the compounds of interest (**1** to **6**) from Autodock 4.2.6 compared to Autodock Vina.

Autodock 4.2.6									Autodock Vina
Compound	vdW+ Hbond (I)	Elec. Energy (II)	Desol. Energy (III)	Total Intermol. Interact. Energy (IV; I + II + III)	Total Internal Energy (V)	Tors. Free Energy (VI)	Unbound’s Energy (VII)	Binding Energy (Kcal/mol) (VIII; IV + V + VI + VII)	Affinity (Kcal/mol)
**Myricetin derivatives**									
**1**	−7.49 *	0.06	5.53	−1.90	−2.05	2.09	0.00	−1.86	−6.6
**2**	−10.43	0.47	5.51	−4.46	−1.16	2.09	0.00	−3.53	−6.0
**5**	51.35 *	0.86	9.30 *	61.50	−1.60	3.88	0.00	63.78	0.0
**Kaempferol derivatives**									
**3**	−7.99	0.26	4.22	−3.50	−1.01	1.49	0.00	−3.02	−5.9
**4**	−9.62 *	−0.10	7.91 *	−1.80	−2.77	3.28	0.00	−1.29	−4.6
**6b**	47.06 *	0.29	7.62	54.97	−2.78	3.28	0.00	55.47	−4.2
**Other**									
**6a**	−12.98 *	0.23	8.34 *	−4.41	−2.07	3.58	0.00	−2.90	−6.3

vdW + Hbonding = Van der Waals + Hydrogen bonding, Elec. Energy = Electrostatic energy, Desol. Energy = Desolvation energy, Total Intermol. Interact. Energy = Total Intermolecule Interaction energy, Tors. Free energy = Torsion free energy. * indicates a major contribution and distinct difference among isolated flavonoids.

## Data Availability

Data sharing is not applicable.

## Supplementary Materials

The following are available online at https://www.mdpi.com/article/10.3390/molecules27238189/s1, Figure S1. The HPLC chromatogram of compound 6 detected at λ_max_ 260, 292 and 370 nm. Figure S2. Three-dimensional structural alignment of all docked flavonoids. (A) Multiple structural alignments of compound **1,** red; compound **2**, white; and compound **5**, orange. (B) Pair structural alignment of compound **1**, red, and compound **2**, white. (C) Pair structural alignment of compound **1**, red, and compound **5**, orange. (D) Multiple structural alignments of compound **3**, green; compound **4**, yellow; and compound **6b**, purple. (E) Pair structural alignment of compound **3**, green, and compound **6b**, purple. (F) Pair structural alignment of compound **6b**, purple, and compound **4**, yellow. Figure S3. Pair structural alignment of compound **6b**, purple, and compound **6a**, blue.

## Sample Availability

Samples of the extracts and compounds are available from the authors.
