# Supplementary material for: Flavonoid Constituents and Alpha-Glucosidase Inhibition of Solanum stramonifolium Jacq. Inflorescence with In Vitro and In Silico Studies
Source: Molecules. 2022 Nov 24;27(23):8189. doi: 10.3390/molecules27238189 (PMC9736281; doi:10.3390/molecules27238189)
Supplement: Supplementary file 1 [file molecules-27-08189-s001.zip › molecules-2021878-supplementary.pdf]

Supplementary Materials

Flavonoid Constituents and Alpha-Glucosidase Inhibition of *Solanum stramonifolium* Jacq. Inflorescence with *In vitro* and *In Silico* Studies

Sukanya Dej-adisai [1,*], Oraphan Sakulkeo [1], Chatchai Wattanapiromsakul [1] and Thanet Pitakbut [2,3]

Contents

Figure S1. The HPLC chromatogram of compound 6 detected at λ_{max} 260, 292 and 370 nm.

Figure S2. 3D Structural alignment of all docked flavonoids. (A) multiple structural alignments of compound **1,** red, compound **2**, white, and compound **5**, orange. (B) pair structural alignment of compound **1**, red, and compound **2**, white. (C) pair structural alignment of compound **1**, red, and compound **5**, orange. (D) multiple structural alignments of compound **3**, green, compound **4**, yellow, and compound **6b**, purple. (E) pair structural alignment of compound **3**, green, and compound **6b**, purple. (F) pair structural alignment of compound **6b**, purple, and compound **4**, yellow.

Figure S3. Pair structural alignment of compound **6b**, purple, and compound **6a**, blue.

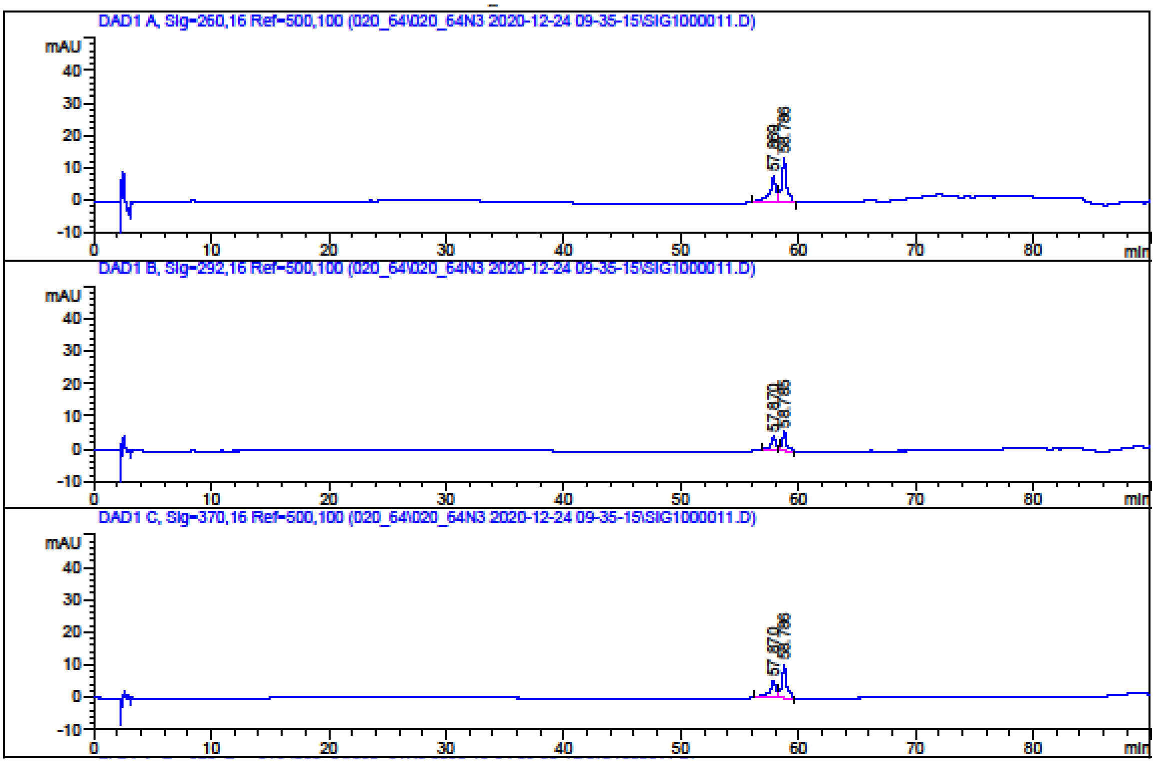

Figure S1. The HPLC chromatogram of compound 6 detected at λ_{max} 260, 292 and 370 nm.

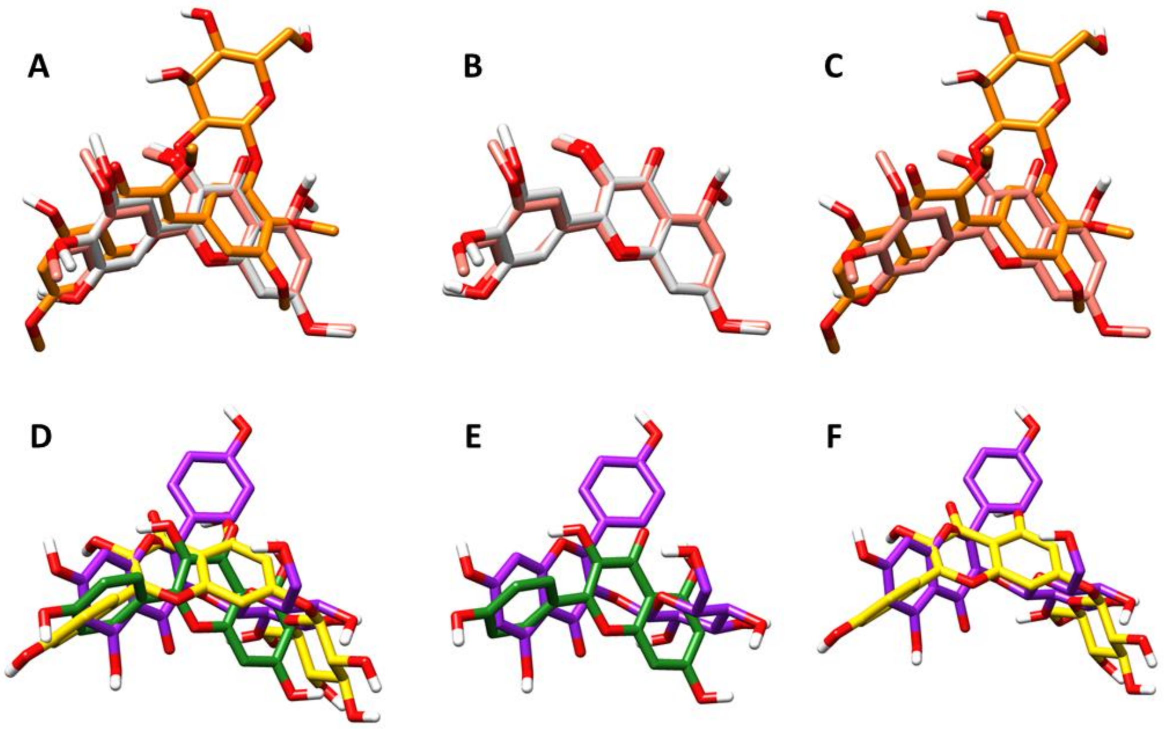

Figure S2. 3D Structural alignment of all docked flavonoids. (A) multiple structural alignments of compound **1,** red, compound **2**, white, and compound **5**, orange. (B) pair structural alignment of compound **1**, red, and compound **2**, white. (C) pair structural alignment of compound **1**, red, and compound **5**, orange. (D) multiple structural alignments of compound **3**, green, compound **4**, yellow, and compound **6b**, purple. (E) pair structural alignment of compound **3**, green, and compound **6b**, purple. (F) pair structural alignment of compound **6b**, purple, and compound **4**, yellow.

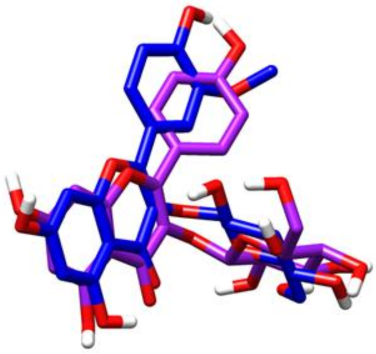

Figure S3. Pair structural alignment of compound **6b**, purple, and compound **6a**, blue.
